# Natural perovskite: (Ca^II^_0.95 (1)_Ce^III^_0.011 (2)_Na^I^_0.010 (4)_)(Fe^III^_0.022 (2)_Ti^IV^_0.98 (1)_)O_3_

**DOI:** 10.1107/S1600536808026421

**Published:** 2008-08-30

**Authors:** Érica G. Gravina, José D. Ayala, Nelson G. Fernandes

**Affiliations:** a Department of Chemistry, Federal University of Minas Gerais, Av. Antônio Carlos 6627, 31270-901 Belo Horizonte, Brazil

## Abstract

A natural sample of perovskite (calcium caesium sodium iron titanium oxide) from the Tapira Alkaline Complex in southeastern Brazil was found by electron microprobe analysis to have the chemical formula (Ca^2+^_0.95 (1)_Ce^3+^_0.011 (2)_Na^+^_0.010 (4)_)(Fe^3+^_0.022 (2)_Ti^4+^_0.98 (1)_)O^2−^_3_ and by IR spectroscopy to be an anhydrous mineral. Oxygen anions are arranged around Ti^4+^ in an almost perfect octa­hedron and around Ca^2+^ in a distorted 12-fold polyhedron.

## Related literature

For related literature, see: Banfield & Veblen (1992 ▶); Beran *et al.* (1996 ▶); Chakhmouradian & Mitchell (1998 ▶); Haggerty & Mariano (1983 ▶); Kay & Bailey (1957 ▶); Lloyd & Bailey (1991 ▶); Mariano & Mitchell (1991 ▶); Seer & Moraes (1988 ▶); Sgarbi & Gaspar (1995 ▶); Sgarbi & Valença (1994 ▶); Soubies *et al.* (1991 ▶).

## Experimental

### 

#### Crystal data

Na_0.01_Ca_0.96_Fe_0.02_Ti_0.98_Ce_0.01_O_3_
*M* *_r_* = 136.40
Orthorhombic, 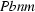

*a* = 5.3818 (4) Å
*b* = 5.4431 (4) Å
*c* = 7.6450 (5) Å
*V* = 223.95 (3) Å^3^
*Z* = 4
Mo *K*α radiation
μ = 5.94 mm^−1^
*T* = 298 (2) K
0.2 × 0.15 × 0.15 mm

#### Data collection

Siemens P4 diffractometer
Absorption correction: refined from Δ*F* (*SHELXL97*; Sheldrick, 2008 ▶) *T*_min_ = 0.356, *T*_max_ = 0.409
2383 measured reflections
1594 independent reflections
1527 reflections with *I* > 2s(*I*)
*R*_int_ = 0.033
3 standard reflections every 197 reflections intensity decay: 0.8%

#### Refinement

*R*[*F* ^2^ > 2σ(*F* ^2^)] = 0.041
*wR*(*F* ^2^) = 0.103
*S* = 1.25
1594 reflections
31 parameters
Δρ_max_ = 2.01 e Å^−3^
Δρ_min_ = −2.88 e Å^−3^

### 

Data collection: *XSCANS* (Siemens, 1991 ▶); cell refinement: *XSCANS*; data reduction: *XSCANS*; program(s) used to solve structure: *SHELXS97* (Sheldrick, 2008 ▶); program(s) used to refine structure: *SHELXL97* (Sheldrick, 2008 ▶); molecular graphics: *CrystalMaker* (CrystalMaker, 2007 ▶); software used to prepare material for publication: *WinGX* (Farrugia, 1999 ▶).

## Supplementary Material

Crystal structure: contains datablocks global, I. DOI: 10.1107/S1600536808026421/mg2053sup1.cif

Structure factors: contains datablocks I. DOI: 10.1107/S1600536808026421/mg2053Isup2.hkl

Additional supplementary materials:  crystallographic information; 3D view; checkCIF report

## Figures and Tables

**Table 1:** Selected geometric parameters (Å ,° ), where *A* represents the Ca^2+^, Na^+^ and Ce^3+^ cations, on a 12-coordinated site and *B* represents Fe^3+^ and Ti^4+^ cations on an octahedral site

*A*—O_1_	2.359 (2)	O_1_^iv^—A—O_1_^iv^	162.30 (6)
*A*—O_1_^iv^	2.481 (2)	O_2_^iv^—*A*—O_2_^viii^	80.97 (4)
*A*—O_1_^iv^	3.027 (2)	O_1_—*A*—O_2_^vii^	118.03 (2)
*A*—O_1_	3.052 (2)	O_2_^ii^—*A*—O_1_^iv^	65.08 (3)
*A*—O_2_^viii^	2.378 (1)		
*A*—O_2_^ii^	2.620 (1)		
*A*—O_2_^iv^	2.667 (1)		
*A*—O_2_^vi^	3.233 (1)		
*B*—O_1_^ii^	1.9513 (3)	O_1_—*B*—O_1_^ii^	180.0
*B*—O_2_^vii^	1.956 (1)	O_2_—*B*—O_2_^vii^	89.41 (1)
*B*—O_2_^v^	1.959 (1)	O_1_—*B*—O_2_	89.58 (6)

Symmetry code: (ii) 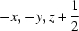; (iii) 
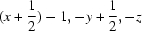
; (iv) 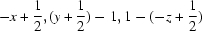; (v) 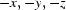; (vi) 
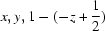
; (vii) 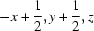; (viii) 
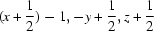
; (ix) 
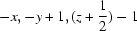
.

**Fig. 1.**
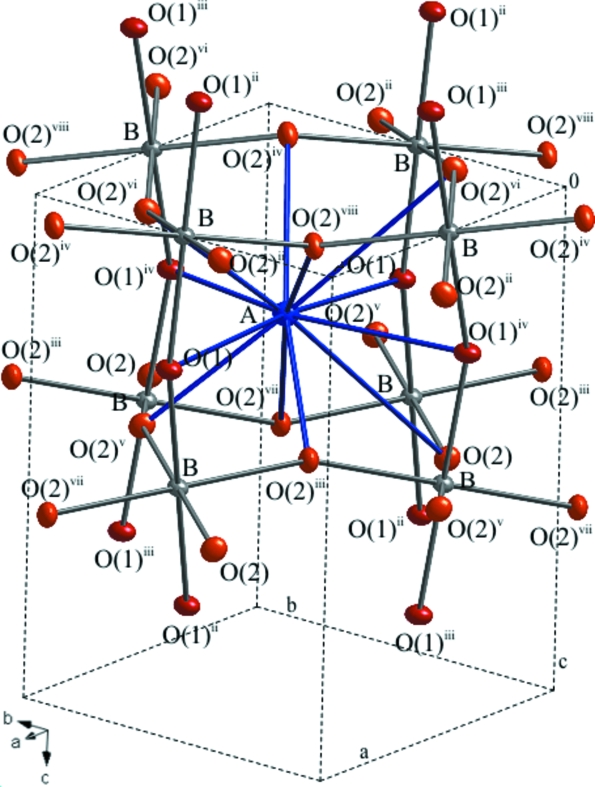
Part of the unit cell with the A site represented in dark blue, the B site in dark grey, and the oxygen ions in dark red. The bonds of the AO12 are also all represented in blue. Displacement ellipsoids are drawn at the 70% probability level [symmetry codes: (i) x, y, z; (ii) -x, -y, z+1/2; (iii) (x+1/2)-1, -y+1/2, -z; (iv) -x+1/2, (y+1/2)-1, 1-(-z+1/2); (v) -x, -y, -z; (vi) x, y, 1-(-z+1/2); (vii) -x+1/2, y+1/2, z; (viii) (x+1/2)-1, -y+1/2, z+1/2; (ix) -x, -y+1, (z+1/2)-1].

## supplementary crystallographic information

### Comment

The term perovskite refers to both natural and synthetic compounds ABX_3_ based on the mineral CaTiO_3_. CaTiO_3_ itself is the most commonly occurring perovskite in the Earth's crust (Chakhmouradian & Mitchell, 1998) and an important material to immobilize high-level radioactive waste. The first structure determination was reported for a synthetic material (Kay & Bailey, 1957), but structural studies of natural CaTiO_3_ are quite rare and have been mostly limited to twinned crystals (Beran *et al.*, 1996). In central and southeastern Brazil, perovskite can be found as essential and accessory minerals of the Alto Paranaíba Igneous Province (Seer & Moraes, 1988; Sgarbi & Valença, 1994; Sgarbi & Gaspar, 1995), where they form part of five important carbonatite complexes, as in Tapira (Lloyd & Bailey, 1991). In these complexes, the conversion of perovskite in anatase (Soubies *et al.*, 1991) resulted in some of the biggest known titanium concentrations, but even so these deposits are not still economically explored for technological reasons. There are many geological studies (Haggerty & Mariano, 1983; Mariano & Mitchell, 1991; and others) describing crystals of perovskite in the Brazilian carbonatite complexes as belonging to the system lueshite (NaNbO_3_)-loparite [(NaCe)TiO_3_]-perovskite (CaTiO_3_) but with the end member perovskite sensu stricto as the principal component.

In this work, a naturally occurring perovskite from the Tapira Alkaline Complex, localized at Minas Gerais State in Brazil (19°52' south and 46°50' west), has been investigated. The economic importance of this complex is due to the phosphates, titanium, and lanthanide and actinide elements drifts, which were formed by intemperism from primary magmatic rocks. From electron microprobe analyses (major elements: Ca - 38.9 (8) wt% CaO; Ti - 56.6 (9) wt% TiO_2_; minor elements: Na - 0.224 (8) wt% Na_2_O; Fe - 1.2 (1) wt% Fe_2_O_3_; Ce - 1.4 (3) wt% Ce_2_O_3_), it can be concluded that the sample is essentially the mineral CaTiO_3_, with the calculated formula: Ca^2+^_0.96 (2)_ Ce^3+^_0.011 (2)_ Na^+^_0.010 (4)_ Fe^3+^_0.022 (2)_ Ti^4+^_0.98 (1)_ O^2-^_3_. The infrared spectra reveal characteristic bands for Ti-O and Ca-O, but importantly, the absence of bands related to OH^-^ and water suggests that the Tapira perovskite is indeed an anhydrous mineral. The bands at 348, 423, 528, 695 and 703 cm^-1^ observed are also present in spectra of TiO_2_ polymorphs, especially anatase and TiO_2_(B) as reported by Banfield & Veblen (1992). This could be due to the octahedral TiO_6_ or even to the Ca^2+^ leaching from the perovskite, which has those two polymorphs as byproducts. Figure 1 shows the perovskite structure.

### Experimental

Among crystals averaging 1-2 cm^3^ in size, some have carbonate incrustations and alterations due to intemperism. The cleanest crystals were separated and the biggest were chosen for polished sections for chemical analysis. Electron microprobe analyses were performed for four crystals on a JEOL JXA-8900 RL microscope, qualitatively with wavelength-dispersive mode and quantitatively with energy-dispersive mode. Standards used included rutile (TiO_2_) for Ti, anorthite (CaAl_2_Si_2_O_8_) for Ca, olivine [(Mg,Fe)_2_SiO_4_] for Fe, albite (NaAlSi_3_O_8_) for Na, and synthetic glasses for the lanthanide content. Infrared spectra were recorded for ground crystals on a Perkin Elmer GX spectrophotometer. Crystals were examined by polarizing microscope.

Initial refinements were performed using scattering factors for the neutral atoms of the major elements, with site occupancies based on the microprobe analyses (Ca_0.96 (2)_Ti_0.98 (1)_O_3_), giving *R* = 0.0436, *wR* = 0.1099, and S = 1.315. The minor elements were added next, with the cation distribution based on the loparite (Na and Ce at A site) and latrappite structures (Fe at B site), and with the constraints that the displacement parameters of atoms within each of these sites be equal. The site occupancies for Ca and Ti were refined whereas those for the Na, Ce, and Fe atoms (including their uncertainties) were taken from the chemical analysis. In the final model, scattering factors for the ions were used and electroneutrality was found to be maintained with a total cation charge of +5.89 (2), according to the chemical formula (Ca^2+^_0.95 (1)_ Ce^3+^_0.011 (2)_ Na^+^_0.010 (4)_) (Fe^3+^_0.022 (2)_Ti^4+^_0.98 (1)_)O^2-^_3_.

### Crystal data

**Table:**

Na_0.01_Ca_0.96_Fe_0.02_Ti_0.98_Ce_0.01_O_3_	*F*_000_ = 262.9
*M**_r_* = 136.40	*D*_x_ = 4.045 Mg m^−^^3^
Orthorhombic, *P**b**n**m*	Mo *K*α radiation λ = 0.71073 Å
Hall symbol: -P 2c 2ab	Cell parameters from 40 reflections
*a* = 5.3818 (4) Å	θ = 4.6–56.8º
*b* = 5.4431 (4) Å	µ = 5.94 mm^−^^1^
*c* = 7.6450 (5) Å	*T* = 298 (2) K
*V* = 223.95 (3) Å^3^	Octahedral, grey
*Z* = 4	0.2 × 0.15 × 0.15 mm

### Data collection

**Table:**

Siemens P4 diffractometer	*R*_int_ = 0.033
Radiation source: fine-focus sealed tube	θ_max_ = 56.8º
Monochromator: graphite	θ_min_ = 4.6º
*T* = 298 K	*h* = −1→12
θ/2θ scans	*k* = −1→12
Absorption correction: part of the refinement model (Δ*F*)(SHELXL97; Sheldrick, 2008)	*l* = −1→18
*T*_min_ = 0.356, *T*_max_ = 0.409	3 standard reflections
2383 measured reflections	every 197 reflections
1594 independent reflections	intensity decay: 0.8%
1527 reflections with *I* > 2s(*I*)	

### Refinement

**Table:**

Refinement on *F*^2^	Secondary atom site location: difference Fourier map
Least-squares matrix: full	*w* = 1/[σ^2^(*F*_o_^2^) + (0.0192*P*)^2^ + 1.1174*P*] where *P* = (*F*_o_^2^ + 2*F*_c_^2^)/3
*R*[*F*^2^ > 2σ(*F*^2^)] = 0.041	(Δ/σ)_max_ < 0.001
*wR*(*F*^2^) = 0.103	Δρ_max_ = 2.01 e Å^−^^3^
*S* = 1.25	Δρ_min_ = −2.88 e Å^−^^3^
1594 reflections	Extinction correction: SHELXL97 (Sheldrick, 2008), Fc^*^=kFc[1+0.001xFc^2^λ^3^/sin(2θ)]^-1/4^
31 parameters	Extinction coefficient: 0.045 (5)
Primary atom site location: structure-invariant direct methods	

### Special details

**Table:**

Experimental. Room temperature single-crystal X-ray diffraction standard experiment
Geometry. All e.s.d.'s (except the e.s.d. in the dihedral angle between two l.s. planes) are estimated using the full covariance matrix. The cell e.s.d.'s are taken into account individually in the estimation of e.s.d.'s in distances, angles and torsion angles; correlations between e.s.d.'s in cell parameters are only used when they are defined by crystal symmetry. An approximate (isotropic) treatment of cell e.s.d.'s is used for estimating e.s.d.'s involving l.s. planes.
Refinement. Refinement of *F*^2^ against ALL reflections. The weighted *R*-factor *wR* and goodness of fit *S* are based on *F*^2^, conventional *R*-factors *R* are based on *F*, with *F* set to zero for negative *F*^2^. The threshold expression of *F*^2^ > σ(*F*^2^) is used only for calculating *R*-factors(gt) *etc*. and is not relevant to the choice of reflections for refinement. *R*-factors based on *F*^2^ are statistically about twice as large as those based on *F*, and *R*- factors based on ALL data will be even larger.

### Fractional atomic coordinates and isotropic or equivalent isotropic displacement parameters (Å^2^)

**Table:**

	*x*	*y*	*z*	*U*_iso_*/*U*_eq_	Occ. (<1)
Ca	0.50660 (7)	0.53492 (7)	0.2500	0.00739 (7)	0.951 (7)
Ce	0.50660 (7)	0.53492 (7)	0.2500	0.0074 (11)	0.01
Na	0.50660 (7)	0.53492 (7)	0.2500	0.0074 (11)	0.01
Ti	0.0000	0.5000	0.5000	0.00487 (6)	0.977 (7)
Fe	0.0000	0.5000	0.5000	0.0049 (11)	0.02
O1	0.0713 (3)	0.4842 (3)	0.2500	0.00743 (19)	
O2	0.21101 (17)	0.21143 (18)	0.53714 (14)	0.00728 (15)	

### Atomic displacement parameters (Å^2^)

**Table:**

	*U*^11^	*U*^22^	*U*^33^	*U*^12^	*U*^13^	*U*^23^
Ca	0.00587 (16)	0.00800 (13)	0.00831 (13)	0.00138 (8)	0.000	0.000
Ce	0.006 (3)	0.00800 (16)	0.00831 (13)	0.00138 (13)	0.000	0.000
Na	0.006 (3)	0.00800 (16)	0.00831 (13)	0.00138 (13)	0.000	0.000
Ti	0.00482 (11)	0.00595 (10)	0.00384 (9)	0.00000 (6)	−0.00004 (6)	−0.00027 (6)
Fe	0.005 (3)	0.00595 (11)	0.00384 (10)	0.00000 (6)	−0.00004 (6)	−0.00027 (9)
O1	0.0072 (4)	0.0104 (4)	0.0047 (4)	−0.0005 (3)	0.000	0.000
O2	0.0060 (3)	0.0072 (3)	0.0087 (3)	0.0020 (2)	0.0005 (2)	0.0010 (2)

### Geometric parameters (Å, °)

**Table:**

Ca—O1	2.3586 (16)	Fe—O2^iii^	1.9555 (9)
Ca—O2^i^	2.3783 (11)	Fe—O2^x^	1.9555 (9)
Ca—O2^ii^	2.3783 (11)	Fe—O2^ix^	1.9589 (9)
Ca—O1^iii^	2.4814 (16)	Fe—O2	1.9589 (9)
Ca—O2^iv^	2.6199 (11)	Fe—Ce^xi^	3.1721 (4)
Ca—O2^v^	2.6199 (11)	Fe—Ca^xi^	3.1721 (4)
Ca—O2^vi^	2.6671 (11)	Fe—Ce^vii^	3.1721 (4)
Ca—O2^iii^	2.6671 (11)	Fe—Ca^vii^	3.1721 (4)
Ca—O1^vii^	3.0266 (16)	Fe—Ca^xii^	3.2772 (3)
Ca—O1^viii^	3.0518 (16)	Fe—Ce^xii^	3.2772 (3)
Ti—O1	1.9513 (3)	O1—Fe^xiii^	1.9513 (3)
Ti—O1^ix^	1.9513 (3)	O1—Ti^xiii^	1.9513 (3)
Ti—O2^iii^	1.9555 (9)	O1—Ca^vii^	2.4814 (16)
Ti—O2^x^	1.9555 (9)	O1—Ce^vii^	2.4814 (16)
Ti—O2^ix^	1.9589 (9)	O1—Ca^iii^	3.0266 (16)
Ti—O2	1.9589 (9)	O1—Ca^xii^	3.0518 (16)
Ti—Ce^xi^	3.1721 (4)	O2—Fe^ii^	1.9555 (9)
Ti—Ca^xi^	3.1721 (4)	O2—Ti^ii^	1.9555 (9)
Ti—Ce^vii^	3.1721 (4)	O2—Ca^x^	2.3783 (11)
Ti—Ca^vii^	3.1721 (4)	O2—Ce^x^	2.3783 (11)
Ti—Ca^xii^	3.2772 (3)	O2—Ca^iv^	2.6199 (11)
Ti—Ce^xii^	3.2772 (3)	O2—Ce^iv^	2.6199 (11)
Fe—O1	1.9513 (3)	O2—Ce^vii^	2.6671 (11)
Fe—O1^ix^	1.9513 (3)	O2—Ca^vii^	2.6671 (11)
			
O1—Ca—O2^i^	113.17 (4)	O2^ix^—Fe—O2	180.0
O1—Ca—O2^ii^	113.17 (4)	O1—Fe—Ce^xi^	128.54 (5)
O2^i^—Ca—O2^ii^	86.35 (5)	O1^ix^—Fe—Ce^xi^	51.46 (5)
O1—Ca—O1^iii^	86.98 (4)	O2^iii^—Fe—Ce^xi^	55.53 (3)
O2^i^—Ca—O1^iii^	129.34 (3)	O2^x^—Fe—Ce^xi^	124.47 (3)
O2^ii^—Ca—O1^iii^	129.34 (3)	O2^ix^—Fe—Ce^xi^	56.90 (3)
O1—Ca—O2^iv^	129.62 (3)	O2—Fe—Ce^xi^	123.10 (3)
O2^i^—Ca—O2^iv^	116.99 (3)	O1—Fe—Ca^xi^	128.54 (5)
O2^ii^—Ca—O2^iv^	66.66 (2)	O1^ix^—Fe—Ca^xi^	51.46 (5)
O1^iii^—Ca—O2^iv^	65.08 (3)	O2^iii^—Fe—Ca^xi^	55.53 (3)
O1—Ca—O2^v^	129.62 (3)	O2^x^—Fe—Ca^xi^	124.47 (3)
O2^i^—Ca—O2^v^	66.66 (2)	O2^ix^—Fe—Ca^xi^	56.90 (3)
O2^ii^—Ca—O2^v^	116.99 (3)	O2—Fe—Ca^xi^	123.10 (3)
O1^iii^—Ca—O2^v^	65.08 (3)	O1—Fe—Ce^vii^	51.46 (5)
O2^iv^—Ca—O2^v^	76.80 (5)	O1^ix^—Fe—Ce^vii^	128.54 (5)
O1—Ca—O2^vi^	66.80 (3)	O2^iii^—Fe—Ce^vii^	124.47 (3)
O2^i^—Ca—O2^vi^	80.97 (4)	O2^x^—Fe—Ce^vii^	55.53 (3)
O2^ii^—Ca—O2^vi^	165.78 (3)	O2^ix^—Fe—Ce^vii^	123.10 (3)
O1^iii^—Ca—O2^vi^	64.58 (3)	O2—Fe—Ce^vii^	56.90 (3)
O2^iv^—Ca—O2^vi^	125.177 (19)	Ce^xi^—Fe—Ce^vii^	180.0
O2^v^—Ca—O2^vi^	63.491 (11)	Ca^xi^—Fe—Ce^vii^	180.0
O1—Ca—O2^iii^	66.80 (3)	O1—Fe—Ca^vii^	51.46 (5)
O2^i^—Ca—O2^iii^	165.78 (3)	O1^ix^—Fe—Ca^vii^	128.54 (5)
O2^ii^—Ca—O2^iii^	80.97 (4)	O2^iii^—Fe—Ca^vii^	124.47 (3)
O1^iii^—Ca—O2^iii^	64.58 (3)	O2^x^—Fe—Ca^vii^	55.53 (3)
O2^iv^—Ca—O2^iii^	63.491 (11)	O2^ix^—Fe—Ca^vii^	123.10 (3)
O2^v^—Ca—O2^iii^	125.177 (19)	O2—Fe—Ca^vii^	56.90 (3)
O2^vi^—Ca—O2^iii^	110.78 (5)	Ce^xi^—Fe—Ca^vii^	180.0
O1—Ca—O1^vii^	75.32 (5)	Ca^xi^—Fe—Ca^vii^	180.0
O2^i^—Ca—O1^vii^	60.38 (3)	O1—Fe—Ca^xii^	65.84 (4)
O2^ii^—Ca—O1^vii^	60.38 (3)	O1^ix^—Fe—Ca^xii^	114.16 (4)
O1^iii^—Ca—O1^vii^	162.30 (6)	O2^iii^—Fe—Ca^xii^	134.03 (3)
O2^iv^—Ca—O1^vii^	127.03 (3)	O2^x^—Fe—Ca^xii^	45.97 (3)
O2^v^—Ca—O1^vii^	127.03 (3)	O2^ix^—Fe—Ca^xii^	53.08 (3)
O2^vi^—Ca—O1^vii^	107.21 (3)	O2—Fe—Ca^xii^	126.92 (3)
O2^iii^—Ca—O1^vii^	107.21 (3)	Ce^xi^—Fe—Ca^xii^	108.311 (8)
O1—Ca—O1^viii^	168.10 (7)	Ca^xi^—Fe—Ca^xii^	108.311 (8)
O2^i^—Ca—O1^viii^	59.23 (3)	Ce^vii^—Fe—Ca^xii^	71.689 (8)
O2^ii^—Ca—O1^viii^	59.23 (3)	Ca^vii^—Fe—Ca^xii^	71.689 (8)
O1^iii^—Ca—O1^viii^	104.92 (5)	O1—Fe—Ce^xii^	65.84 (4)
O2^iv^—Ca—O1^viii^	57.99 (3)	O1^ix^—Fe—Ce^xii^	114.16 (4)
O2^v^—Ca—O1^viii^	57.99 (3)	O2^iii^—Fe—Ce^xii^	134.03 (3)
O2^vi^—Ca—O1^viii^	118.03 (2)	O2^x^—Fe—Ce^xii^	45.97 (3)
O2^iii^—Ca—O1^viii^	118.03 (2)	O2^ix^—Fe—Ce^xii^	53.08 (3)
O1^vii^—Ca—O1^viii^	92.78 (5)	O2—Fe—Ce^xii^	126.92 (3)
O1—Ti—O1^ix^	180.0	Ce^xi^—Fe—Ce^xii^	108.311 (8)
O1—Ti—O2^iii^	90.66 (5)	Ca^xi^—Fe—Ce^xii^	108.311 (8)
O1^ix^—Ti—O2^iii^	89.34 (5)	Ce^vii^—Fe—Ce^xii^	71.689 (8)
O1—Ti—O2^x^	89.34 (5)	Ca^vii^—Fe—Ce^xii^	71.689 (8)
O1^ix^—Ti—O2^x^	90.66 (5)	Fe^xiii^—O1—Fe	156.74 (9)
O2^iii^—Ti—O2^x^	180.0	Ti^xiii^—O1—Fe	156.74 (9)
O1—Ti—O2^ix^	90.42 (6)	Fe^xiii^—O1—Ti	156.74 (9)
O1^ix^—Ti—O2^ix^	89.58 (6)	Ti^xiii^—O1—Ti	156.74 (9)
O2^iii^—Ti—O2^ix^	90.586 (13)	Fe^xiii^—O1—Ca	100.97 (4)
O2^x^—Ti—O2^ix^	89.414 (13)	Ti^xiii^—O1—Ca	100.97 (4)
O1—Ti—O2	89.58 (6)	Fe—O1—Ca	100.97 (4)
O1^ix^—Ti—O2	90.42 (6)	Ti—O1—Ca	100.97 (4)
O2^iii^—Ti—O2	89.414 (13)	Fe^xiii^—O1—Ca^vii^	90.58 (5)
O2^x^—Ti—O2	90.586 (13)	Ti^xiii^—O1—Ca^vii^	90.58 (5)
O2^ix^—Ti—O2	180.0	Fe—O1—Ca^vii^	90.58 (5)
O1—Ti—Ce^xi^	128.54 (5)	Ti—O1—Ca^vii^	90.58 (5)
O1^ix^—Ti—Ce^xi^	51.46 (5)	Ca—O1—Ca^vii^	106.45 (6)
O2^iii^—Ti—Ce^xi^	55.53 (3)	Fe^xiii^—O1—Ce^vii^	90.58 (5)
O2^x^—Ti—Ce^xi^	124.47 (3)	Ti^xiii^—O1—Ce^vii^	90.58 (5)
O2^ix^—Ti—Ce^xi^	56.90 (3)	Fe—O1—Ce^vii^	90.58 (5)
O2—Ti—Ce^xi^	123.10 (3)	Ti—O1—Ce^vii^	90.58 (5)
O1—Ti—Ca^xi^	128.54 (5)	Ca—O1—Ce^vii^	106.45 (6)
O1^ix^—Ti—Ca^xi^	51.46 (5)	Fe^xiii^—O1—Ca^iii^	85.94 (5)
O2^iii^—Ti—Ca^xi^	55.53 (3)	Ti^xiii^—O1—Ca^iii^	85.94 (5)
O2^x^—Ti—Ca^xi^	124.47 (3)	Fe—O1—Ca^iii^	85.94 (5)
O2^ix^—Ti—Ca^xi^	56.90 (3)	Ti—O1—Ca^iii^	85.94 (5)
O2—Ti—Ca^xi^	123.10 (3)	Ca—O1—Ca^iii^	91.25 (5)
O1—Ti—Ce^vii^	51.46 (5)	Ca^vii^—O1—Ca^iii^	162.30 (6)
O1^ix^—Ti—Ce^vii^	128.54 (5)	Ce^vii^—O1—Ca^iii^	162.30 (6)
O2^iii^—Ti—Ce^vii^	124.47 (3)	Fe^xiii^—O1—Ca^xii^	78.47 (4)
O2^x^—Ti—Ce^vii^	55.53 (3)	Ti^xiii^—O1—Ca^xii^	78.47 (4)
O2^ix^—Ti—Ce^vii^	123.10 (3)	Fe—O1—Ca^xii^	78.47 (4)
O2—Ti—Ce^vii^	56.90 (3)	Ti—O1—Ca^xii^	78.47 (4)
Ce^xi^—Ti—Ce^vii^	180.0	Ca—O1—Ca^xii^	168.10 (7)
Ca^xi^—Ti—Ce^vii^	180.0	Ca^vii^—O1—Ca^xii^	85.45 (4)
O1—Ti—Ca^vii^	51.46 (5)	Ce^vii^—O1—Ca^xii^	85.45 (4)
O1^ix^—Ti—Ca^vii^	128.54 (5)	Ca^iii^—O1—Ca^xii^	76.85 (4)
O2^iii^—Ti—Ca^vii^	124.47 (3)	Fe^ii^—O2—Ti	155.77 (6)
O2^x^—Ti—Ca^vii^	55.53 (3)	Ti^ii^—O2—Ti	155.77 (6)
O2^ix^—Ti—Ca^vii^	123.10 (3)	Fe^ii^—O2—Fe	155.77 (6)
O2—Ti—Ca^vii^	56.90 (3)	Ti^ii^—O2—Fe	155.77 (6)
Ce^xi^—Ti—Ca^vii^	180.0	Fe^ii^—O2—Ca^x^	97.78 (4)
Ca^xi^—Ti—Ca^vii^	180.0	Ti^ii^—O2—Ca^x^	97.78 (4)
O1—Ti—Ca^xii^	65.84 (4)	Ti—O2—Ca^x^	106.45 (4)
O1^ix^—Ti—Ca^xii^	114.16 (4)	Fe—O2—Ca^x^	106.45 (4)
O2^iii^—Ti—Ca^xii^	134.03 (3)	Fe^ii^—O2—Ce^x^	97.78 (4)
O2^x^—Ti—Ca^xii^	45.97 (3)	Ti^ii^—O2—Ce^x^	97.78 (4)
O2^ix^—Ti—Ca^xii^	53.08 (3)	Ti—O2—Ce^x^	106.45 (4)
O2—Ti—Ca^xii^	126.92 (3)	Fe—O2—Ce^x^	106.45 (4)
Ce^xi^—Ti—Ca^xii^	108.311 (8)	Fe^ii^—O2—Ca^iv^	86.50 (4)
Ca^xi^—Ti—Ca^xii^	108.311 (8)	Ti^ii^—O2—Ca^iv^	86.50 (4)
Ce^vii^—Ti—Ca^xii^	71.689 (8)	Ti—O2—Ca^iv^	90.22 (4)
Ca^vii^—Ti—Ca^xii^	71.689 (8)	Fe—O2—Ca^iv^	90.22 (4)
O1—Ti—Ce^xii^	65.84 (4)	Ca^x^—O2—Ca^iv^	98.07 (4)
O1^ix^—Ti—Ce^xii^	114.16 (4)	Ce^x^—O2—Ca^iv^	98.07 (4)
O2^iii^—Ti—Ce^xii^	134.03 (3)	Fe^ii^—O2—Ce^iv^	86.50 (4)
O2^x^—Ti—Ce^xii^	45.97 (3)	Ti^ii^—O2—Ce^iv^	86.50 (4)
O2^ix^—Ti—Ce^xii^	53.08 (3)	Ti—O2—Ce^iv^	90.22 (4)
O2—Ti—Ce^xii^	126.92 (3)	Fe—O2—Ce^iv^	90.22 (4)
Ce^xi^—Ti—Ce^xii^	108.311 (8)	Ca^x^—O2—Ce^iv^	98.07 (4)
Ca^xi^—Ti—Ce^xii^	108.311 (8)	Ce^x^—O2—Ce^iv^	98.07 (4)
Ce^vii^—Ti—Ce^xii^	71.689 (8)	Fe^ii^—O2—Ce^vii^	91.02 (4)
Ca^vii^—Ti—Ce^xii^	71.689 (8)	Ti^ii^—O2—Ce^vii^	91.02 (4)
O1—Fe—O1^ix^	180.0	Ti—O2—Ce^vii^	85.12 (4)
O1—Fe—O2^iii^	90.66 (5)	Fe—O2—Ce^vii^	85.12 (4)
O1^ix^—Fe—O2^iii^	89.34 (5)	Ca^x^—O2—Ce^vii^	99.03 (4)
O1—Fe—O2^x^	89.34 (5)	Ce^x^—O2—Ce^vii^	99.03 (4)
O1^ix^—Fe—O2^x^	90.66 (5)	Ca^iv^—O2—Ce^vii^	162.90 (4)
O2^iii^—Fe—O2^x^	180.0	Ce^iv^—O2—Ce^vii^	162.90 (4)
O1—Fe—O2^ix^	90.42 (6)	Fe^ii^—O2—Ca^vii^	91.02 (4)
O1^ix^—Fe—O2^ix^	89.58 (6)	Ti^ii^—O2—Ca^vii^	91.02 (4)
O2^iii^—Fe—O2^ix^	90.586 (13)	Ti—O2—Ca^vii^	85.12 (4)
O2^x^—Fe—O2^ix^	89.414 (13)	Fe—O2—Ca^vii^	85.12 (4)
O1—Fe—O2	89.58 (6)	Ca^x^—O2—Ca^vii^	99.03 (4)
O1^ix^—Fe—O2	90.42 (6)	Ce^x^—O2—Ca^vii^	99.03 (4)
O2^iii^—Fe—O2	89.414 (13)	Ca^iv^—O2—Ca^vii^	162.90 (4)
O2^x^—Fe—O2	90.586 (13)	Ce^iv^—O2—Ca^vii^	162.90 (4)

Symmetry codes: (i) *x*+1/2, −*y*+1/2, *z*−1/2; (ii) *x*+1/2, −*y*+1/2, −*z*+1; (iii) −*x*+1/2, *y*+1/2, *z*; (iv) −*x*+1, −*y*+1, −*z*+1; (v) −*x*+1, −*y*+1, *z*−1/2; (vi) −*x*+1/2, *y*+1/2, −*z*+1/2; (vii) −*x*+1/2, *y*−1/2, *z*; (viii) *x*+1, *y*, *z*; (ix) −*x*, −*y*+1, −*z*+1; (x) *x*−1/2, −*y*+1/2, −*z*+1; (xi) *x*−1/2, −*y*+3/2, −*z*+1; (xii) *x*−1, *y*, *z*; (xiii) −*x*, −*y*+1, *z*−1/2.

### Table 1 Selected geometric parameters (Å ,° ), where A represents the Ca^2+^, Na^+^ and Ce^3+^ cations, on 12-coordinated site and B represents Fe^3+^ and Ti^4+^ cations on octahedral site

**Table:**

A-O_1_	2.359 (2)	O_1_^iv^-A-O_1_^iv^	162.30 (6)
A-O_1_^iv^	2.481 (2)	O_2_^iv^-A-O_2_^viii^	80.97 (4)
A-O_1_^iv^	3.027 (2)	O_1_-A-O_2_^vii^	118.03 (2)
A-O_1_	3.052 (2)	O_2_^ii^-A-O_1_^iv^	65.08 (3)
A-O_2_^viii^	2.378 (1)		
A-O_2_^ii^	2.620 (1)		
A-O_2_^iv^	2.667 (1)		
A-O_2_^vi^	3.233 (1)		
B-O_1_^ii^	1.9513 (3)	O_1_-B-O_1_^ii^	180.0
B-O_2_^vii^	1.956 (1)	O_2_-B-O_2_^vii^	89.41 (1)
B-O_2_^v^	1.959 (1)	O_1_-B-O_2_	89.58 (6)

Notes: [Symmetry code: i) x, y, z; ii) -x, -y, z + 1/2; iii) (x + 1/2) -1, -y + 1/2, -z; iv) -x + 1/2, (y + 1/2) - 1, 1- (-z + 1/2); v) -x, -y, -z; vi) x, y, 1 - (-z + 1/2); vii) -x + 1/2, y + 1/2, z; viii) (x + 1/2) - 1, -y + 1/2, z + 1/2; ix) -x, -y + 1, (z + 1/2) -1].
